# Middle ear cholesteatoma resection under general anesthesia in patients with congenital methemoglobinemia: A case report

**DOI:** 10.1097/MD.0000000000033561

**Published:** 2023-04-14

**Authors:** Zenghua Cai, Yu Shao, Yu Wu

**Affiliations:** a Department of Anesthesiology, Bethune International Peace Hospital, Shijiazhuang, China.

**Keywords:** cholesteatoma, congenital, general anesthesia, methemoglobinemia, tympanitis

## Abstract

Congenital methemoglobinemia is a rare cyanosis cause that can be manifested in affected individuals. We report a case of congenital methemoglobinemia after middle ear cholesteatoma resection under general anesthesia.

**Patient concerns:**

The primary concern of the patient is to safely perform cholesteatoma resection of the middle ear to reduce the pain associated with years of surgery and to survive the perioperative period.

**Diagnoses:**

Congenital methemoglobinemia type 1.

**Interventions:**

The patient underwent general anesthesia and cholesteatoma resection of the middle ear.

**Outcomes:**

The patient successfully underwent cholesteatoma resection in the middle ear under general anesthesia and went through the perioperative period smoothly, and successfully returned to society.

**Lessons:**

For patients requiring general anesthesia complicated with rare methemoglobinemia, we improve the awareness of crisis and make comprehensive preparation and monitoring, learn the pathophysiological mechanism related to the disease, so as to protect the operation of methemoglobin patients under general anesthesia.

## 1. Introduction

Methemoglobinemia, a rare cause of cyanosis and hypoxemia, may occur in both prior and acquired forms. Congenital methemoglobinemia is a rare familial genetic disease. Due to the defects in the reduction of methemoglobin in cells, the reduction rate of methemoglobin from ferric to ferric is slowed down, resulting in the increase of methemoglobin concentration. Methemoglobinemia is known as methemoglobinemia when the total hemoglobin content is >1.0%. We report a case of middle ear cholesteatoma resection under general anesthesia in a patient with methemoglobinemia with a total hemoglobin content of 25.5% and cholinesterase activity of 100%.

## 2. Case report

A 59-year-old male patient weighing 74 kg was admitted to hospital due to hearing loss in the right ear, intermittent discharge of pus for 2 years, dizziness and ear tightness for 3 months. Two years ago, there was no obvious cause of hearing loss in the right ear and yellow pus flowed. After anti-inflammatory treatment, it was improved. Thereafter, the symptoms of right ear discharge recurred and worsened 3 months ago, accompanied by dizziness and ear tightness. Ultra-thin coronal computed tomography scan of the temporal bone showed right tympanitis, formation of cholesteroma, invasion of the right anterior medial wall of sigmoid sinus and right horizontal semicircular canal. Self-confessed rural farming, no history of chronic diseases such as hypertension and coronary heart disease, no history of food poisoning and exposure to radioactive substances, no history of trauma surgery and blood transfusion, no history of food and drug allergy, no history of tobacco, alcohol and other habits. Physical examination: Temperature 36.2°C, pulse 78 times/minutes, breathing 18 times/minutes, blood pressure 136/80 mm Hg. Normal mental intelligence, no chest tightness, palpitation and dyspnea. Cyanosis of lips, oral mucosa and ends of fingers and toes. The chest radiographs were symmetrical with increased lung texture, small hilum, not wide mediastinum, and normal shape and size of heart shadow. The electrocardiogram (ECG) was generally normal. Left ventricular ejection fraction left ventricular ejection fractions ≈ 65%, short axis shortening rate ≈ 35%, no abnormal wall motion. The spleen was slightly larger, 124 mm in length, 42 mm in intercostal thickness, and there was no abnormality in liver, biliary, pancreas and kidney. White blood cell 5.34 G/L, red blood cells 4.11 G/L, platelet 169 G/L, hemoglobin (Hb) 134 g/L, hematocrit (HCT) 40.1%. total bilirubin 33.3 umol/L, direct Bilirubin 0.7 umol/L, renal function and blood coagulation were normal. The concentration of methemoglobin was 25.5%, no nitrite and other toxic components were detected, and cholinesterase activity was 100%. The diagnosis was: Cholesteatoma tympanitis; Congenital methemoglobinemia. Cholesteatoma resection was performed under general anesthesia.

**Figure 1.**
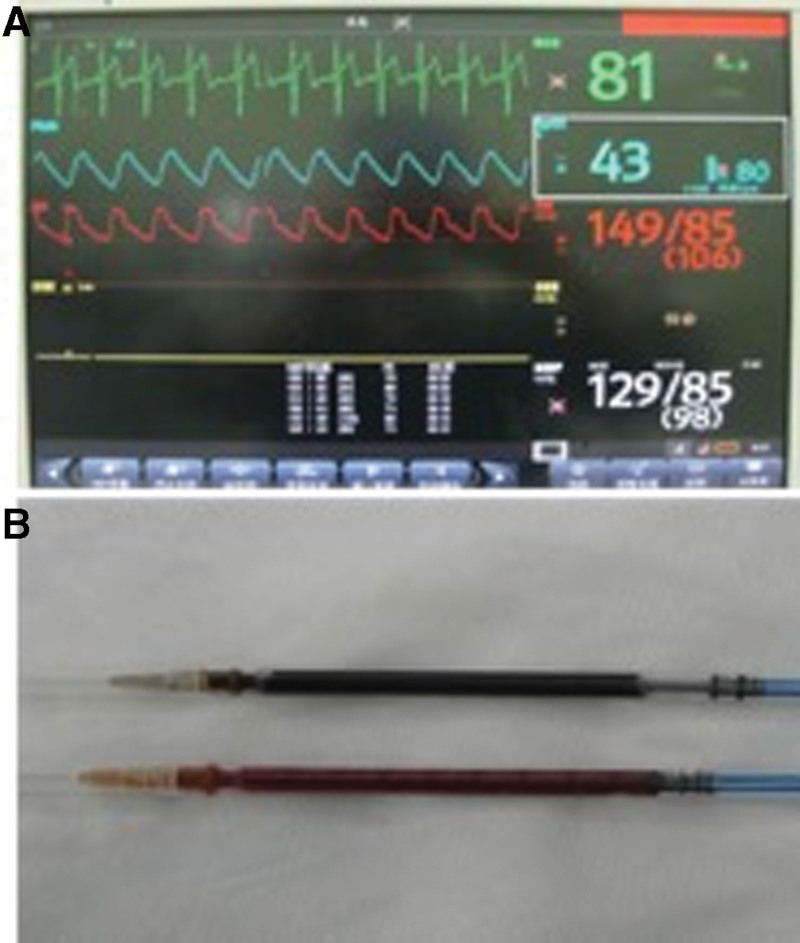
Intraoperative monitoring of patients. (A) The patient’s intraoperative heart rate, heart rate, noninvasive blood pressure, radial artery pressure monitoring, (B) visual comparison of the color of patients radial artery blood.

Diazepam 10 mg and atropine 0.5 mg were intramuscular injections 30 minutes before surgery. ECG, mean arterial pressure and bispectral index (BIS) values were dynamically monitored. Pulse oxygen saturation (SpO_2_) fluctuated between 43% and 47% (Fig. 1). Blood gas analysis without oxygen was performed: PH:7.427, PCO_2_ 41.0mmHg, PO_2_ 75mmHg, HCT 35%, Hb 11.9g/L. Two percentage lidocaine hydrochloride spray was used to anesthetise the mucosal surface of the tongue base and throat, and 3 ml 2% lidocaine hydrochloride was injected to anesthetise the tracheal mucosa through cricothyroid membrane puncture. Meanwhile, 20 mg methylene blue was injected intravenously within 10 minutes according to the literature.^[1–3]^ After successful superficial anesthesia, propofol (2 mg/kg) and atracurium cisbenesulfate (0.15 mg/kg) were administered intravenously, and the tracheal intubation process was smooth and smooth. Mechanical ventilation (tidal volume 8ml/kg, respiratory rate 12 times/min), continuous infusion of propofol 4 mg/(kg·h), reifentanil 0.2 μg/(kg·min), intermittent intravenous infusion of atracurium cissulfonate 0.05 mg/kg to maintain anesthesia, vitamin C 2g static point antioxidant. Intraoperative SpO_2_ fluctuated between 55% and 78%. One hour after anesthesia, blood gas analysis showed PH 7.401, PCO_2_ 45.6mmHg, PO_2_ 283mmHg, HCT 31% and Hb 10.5 g/L. The operation and anesthesia were smooth, and hemodynamics was stable. After the operation, the patient was awake, and the muscle strength and tension were normal. The tracheal catheter was removed, and there were no adverse complaints. The patient returned to the ward and continued monitoring and treatment. There was no abnormality during follow-up, and the patient was discharged from hospital 1 week after surgery.

## 3. Discussion

Congenital methemoglobinemia is caused by gene mutation to decrease the activity or stability of NADH-Cytochrome b5 reductase (b5R),^[4]^ The formation of autosomal recessive genetic disease mainly characterized by methemoglobin (MetHb) reduction disorder.^[5,6]^ There are 2 clinical types: Type I, also known as erythrocyte type, is characterized by decreased stability of b5R, and its clinical manifestations are closely related to the content of MetHb. When the content of MetHb exceeds 10% of the total amount of Hb, cyanosis of lips, oral mucosa and the ends of fingers and toes may occur; when the content of MetHb exceeds 30% of the total amount of Hb, it may cause dyspnea, dizziness and other neurological and respiratory symptoms; when the content exceeds 70% of the total amount of Hb, it may threaten life.^[7]^ Type II, also known as systemic type, is caused by gene mutation to reduce the activity of b5R catalytic capacity,^[8]^ the body cells and nervous system lipid metabolism are involved, clinical manifestations in addition to lip, oral mucosa and the end of the finger and toe cyanosis, often combined with developmental delays, mental and intellectual abnormalities and other neurological symptoms; Most of these patients are children, and most of them die in childhood, live to adult less.

This case is a type I congenital methemoglobin patient with cyanosis of lips, oral mucosa and the end of fingers and toes since childhood, and has a family history.^[9]^ The concentration of MetHb is 25.5%. After mask oxygen inhalation, arterial partial pressure of oxygen increased significantly, but SpO_2_, cyanosis of lip and end of finger and toe did not improve, and blood color was brown.^[10]^ The reason was that MetHb had poor oxygen-carrying and oxygen-releasing capacity, and simple oxygen therapy had little effect on the total hemoglobin oxygen supply, leading to no improvement in the hypoxia state of body tissues and organs. Cyanosis of the lips and ends of the fingers and toes is a typical clinical manifestation of these patients, which is easily misdiagnosed as food or drug poisoning^[11–14]^ and circulatory or respiratory diseases. The diagnosis can be confirmed by inquiring the medical history, combined with clinical manifestations and toxicological screening.

The patient in this case has a long history of otitis media and cholesteatoma, which involves a wide range of diseases and is difficult to operate. General anesthesia is the preferred anesthesia method. Before general anesthesia induction, lidocaine hydrochloride was given surface anesthesia to reduce stress response and the dosage of fentanyl and other opioid analgesics. Select fast-channel anesthetics propofol, remifentanil and atracurium cisphenate all by intravenous anesthesia to reduce the burden of liver and kidney. Methylene blue and vitamin C were used to reduce MetHb before and after anesthesia to increase the oxygen-carrying capacity of Hb and improve the anoxic state of body tissues and organs. After active preparation before surgery, antioxidation symptomatic treatment during perioperative period, the anesthesia and operation process were smooth and smooth, the recovery was timely, and no anesthesia complications occurred. This kind of patient is rare in clinic and inexperienced. When encountering similar patients, our experience is as follows: Improve preoperative examination, fully understand methemoglobin content and lesion degree, and fully evaluate the functions of heart, brain, liver, kidney, and other organs of congenital methemoglobinemia; Methylene blue, vitamin C and other antioxidants as well as fresh allogenic blood input have good therapeutic effects on increasing oxygen-carrying capacity, improving organ function and peripheral cyanosis in these patients; Combined with throat and internal surface anesthesia during general anesthesia can effectively reduce the dosage of fentanyl and other opioids, reduce stress response, reduce oxygen consumption, maintain hemodynamic stability and oxygen supply and demand balance, and protect the function of organs; Monitoring of mean arterial pressure, heart rate, respiration (R), ECG, SpO_2_, BIS (BIS), partial pressure of end-breath carbon dioxide and blood gas analysis was strengthened during the operation. It has a good guiding effect on the development and change of the disease, treatment decision and prognosis.

## Author contributions

**Conceptualization:** Yu Wu.

**Data curation:** Zenghua Cai, Yu Shao.

**Resources:** Yu Shao, Yu Wu.

**Supervision:** Zenghua Cai, Yu Wu.

**Validation:** Zenghua Cai.

**Writing – original draft:** Zenghua Cai.

**Writing – review & editing:** Yu Wu.
